# Response to Esteve-Gassent *et al.*: *flaB* sequences obtained from Texas PCR products are identical to the positive control strain *Borrelia burgdorferi* B31

**DOI:** 10.1186/s13071-015-0899-x

**Published:** 2015-06-09

**Authors:** Steven J. Norris, Alan G. Barbour, Durland Fish, Maria A. Diuk-Wasser

**Affiliations:** Departments of Pathology & Laboratory Medicine and Microbiology and Molecular Genetics, University of Texas Medical School at Houston, PO Box 20708, 77225-0708 Houston, TX USA; Departments of Microbiology and Molecular Genetics, Medicine, and Ecology and Evolutionary Biology, University of California at Irvine, Irvine, USA; Department of Epidemiology of Microbial Diseases, Yale School of Public Health, New Haven, USA; Yale School of Forestry and Environmental Studies, New Haven, USA; Department of Ecology, Evolution and Environmental Sciences, Columbia University, New York, USA

**Keywords:** *Borrelia burgdorferi*, Lyme disease, *Ixodes scapularis*, Texas, Mexico

## Abstract

Feria-Arroyo et al. had reported previously that, based on PCR analysis, 45 % of *Ixodes scapularis* ticks collected in Texas and Mexico were infected with the Lyme disease spirochete *Borrelia burgdorferi* (Parasit. Vectors 2014, **7**:199). However, our analyses of their initial data (Parasit. Vectors 2014, **7**:467) and a recent response by Esteve-Gassent et al. (Parasit. Vectors 2015, **8**:129) provide evidence that the positive PCR results obtained from both ribosomal RNA intergenic sequences and the flagellin gene *flaB* are highly likely due to contamination by the *B. burgdorferi* B31 positive control strain.

In 2014, Feria-Arroyo et al. [1] reported that 45 % of *Ixodes scapularis* ticks collected in Texas and Mexico were infected with the Lyme disease spirochete *Borrelia burgdorferi*, based on nested PCR amplification and sequencing of the 16S rDNA-23S rDNA intergenic spacer region (IGS)*.* Positive PCR results were also reported for the *B. burgdorferi* flagellin gene *flaB*, but no *flaB* sequences were provided in the article. The results reported by Feria-Arroyo et al. [1] were highly questionable in that all prior studies of ticks from Texas indicated that less than two percent were infected with *Borrelia* of any kind. In a comprehensive reanalysis of their data [2], we demonstrated previously that the reported IGS sequences had a high degree of identity with the IGS region of the positive control strain, *B. burgdorferi* B31, used in their study. This commonly used strain was the first isolate of *B. burgdorferi*, and was obtained from ticks collected on Shelter Island, New York in 1981 [3]. Based on our analysis, we concluded that the high frequency of positive PCR results reported by Feria-Arroyo et al. [1] were due to contamination of their tick specimens with DNA from the positive control strain. In a recent response published on 27 February 2015, corresponding author Dr Maria Esteve-Gassent and co-authors [4] provided *flaB* sequences from several of the tick specimens from Texas and claimed that “Infection levels using a second genetic marker (*flaB*), confirmed the results originally obtained by the 16S rRNA-23S rRNA gene intergenic spacer (IGS) of *B. burgdorferi*”. However, a simple comparison of these *flaB* sequences to existing genomic sequences indicated that they are essentially identical to the *B. burgdorferi* B31 sequence, contrary to their conclusions [4]. In addition, the author’s recent response [4] did not address the high sequence identity between the IGS sequences obtained from the tick specimens with that of the positive control strain (see [2]), and contained several additional errors and misstatements. If anything, the additional data and analysis of sequences provided by Esteve-Gassent et al. [4] further strengthen our critique and deepen our doubts about the accuracy of the observations. Therefore, our assessment and conclusions remain the same, namely that: (**1**) The findings reported by Feria-Arroyo and coworkers [1, 4] of *B. burgdorferi* in their sample of ticks from Texas and Mexico are erroneous; and (**2**) The inaccurate information, most plausibly, was the consequence of laboratory contamination of the samples in the chain of possession and faulty analysis of their results and the scientific literature. Our presumption is that the contamination was not intentional but inadvertent. (Indeed, the avoidance of contamination through the scrupulous preparation and handling of specimens is an important and difficult challenge for all laboratories carrying out the highly sensitive PCR procedure [5]). The inaccurate and unreliable information reported is not a trivial matter or of little consequence for public health. Mistaken allegations of the new presence of *B. burgdorferi* in a geographical area, particularly at such high prevalence as Esteve-Gassent and co-authors [1, 4] reported, could lead to clinical misdiagnoses and misdirected prevention, treatment and control efforts.

In particular, we bring attention to the following points.

1. The Esteve-Gassent et al. response does not address the IGS sequence identity between the Texas samples and the positive control strain

Feria-Arroyo et al. [1] concluded that 45 % of *Ixodes scapularis* ticks collected in Texas and Mexico were infected with *Borrelia burgdorferi*. This conclusion was based on positive PCR results obtained with these samples using primers for the 16S rDNA-23S rDNA intergenic spacer region (IGS). In our prior Letter to the Editor [2], we provided clear evidence that the IGS sequences obtained from the Texas specimens had extensive sequence identity with the IGS region with the positive control strain *B. burgdorferi* B31, indicating cross-contamination of the Texas samples with B31 DNA. Extensive regions (664 to 925 bp) were identical to the corresponding B31 sequence in 10 specimens, and were ≥98.9 % identical in the remaining 11 specimens (Table one of [2]). Indeed, the differences from the B31 sequence were concentrated in the 5’ and 3’ regions of the sequences, which appeared to have a high number of sequence errors based on their lack of identity to any *Borrelia* sequences. These points are described in detail in the text and Tables one and two, and Additional file one: Figure S-one of the prior Letter to the Editor by Norris et al. [2]. This crucial aspect was not addressed in the response of Esteve-Gassent et al. [3]. The IGS data are important, in that this intergenic region is much more heterogeneous among *B. burgdorferi* strains than are other regions, including the *flaB* gene. Therefore the extensive IGS sequence identity with the B31 strain is strongly indicative of DNA contamination and false positive results.

2. The *flaB* sequences from the tick samples from Texas are also identical to those of the positive control strain B31

In their response, Esteve-Gassent et al. [4] state that “Infection levels using a second genetic marker (*flaB*), confirmed the results originally obtained by the 16S rRNA-23S rRNA gene intergenic spacer (IGS) of *B. burgdorferi*.” It should first be noted that the flagellin protein gene *flaB* is highly conserved in *B. burgdorferi*, so it is useful for detecting *B. burgdorferi* strains but not for distinguishing between them. Indeed, Figure one in the Esteve-Gassent et al. response [4] indicates a high degree of sequence identity at the nucleotide level (the differences in the sequence of BWTX12-16 are addressed below). In addition, Esteve-Gassent et al. [4] go on to state the following:

“Norris *et al.* argued that the infected ticks reported in our study were found infected with *B. burgdorferi* likely due to contamination of the PCR reactions with DNA from the strain B31 of *B. burgdorferi*, the positive control used in the study. Nevertheless, *B. burgdorferi* B31 *flaB* has a cytosine (C) at position 75 in this alignment (Fig. one) while the Texas isolates had an adenine (A). The A in the Texas isolate makes them more similar to strains N40 and 297 than to B31. Contamination of our samples with strains N40 and 297 is impossible, since these strains are not present in the laboratory in which molecular analyses were carried out”.

We were puzzled by this statement in that the widely accepted *B. burgdorferi* B31 chromosome sequence (GenBank Accession No. AE000783.1) has an adenine (A) at the indicated position. A recent resequencing of the B31 genome (CP009656) has the same sequence. In fact, nearly all of the reported *B. burgdorferi flaB* sequences (over 100) have an adenine at this position. The only exceptions are three reported sequences: two from Dr. Reinhardt Wallich in Germany (X15661 and X16833) and one from a group in the United Kingdom (Y15088). Because the sequence difference is restricted to this one nucleotide, the particular clone used by these groups likely had a point mutation at this position.

Esteve-Gassent et al. [4] apparently selected this rare sequence because the GenBank entry X15661 is annotated as *flaB*. In the B31 genomic sequence, the gene is annotated as *BB0147* and the descriptor used is “P41”, one of the initial descriptions of this protein as a 41-kDa protein antigen. Esteve-Gassent et al. [4] apparently did not recognize that *BB0147* is the same as *flaB*, although a simple BLAST search would have demonstrated this fact.

Esteve-Gassent et al. [4] have deposited 8 *flaB* sequences from Texas samples in the GenBank database; three sequences included in their response (GE64, MM68 and MM161) have not been provided to GenBank. These GenBank sequences are longer than those provided in Fig. one of their response, and are 234 to 238 nt in length. An alignment of the GenBank sequences with the corresponding *flaB* region from the *B. burgdorferi* B31 genome sequence (nt 147949–148187) is shown in Fig. 1. This alignment demonstrates that the *flaB* sequences reported by Esteve-Gassent et al. [4] are identical to the B31 *flaB* gene, with the exception of a few differences (apparent sequence errors) at the ends of their sequences and one near the middle of one of their sequences (MMWMA69-70). The comparable sequences from *B. burgdorferi* N40 and 297 and the GenBank entry from Dr Wallich’s group (X15661) are shown at the bottom of the alignment. The position of the cytosine in question (nt 67) in X15661 is indicated.

**Fig. 1:**
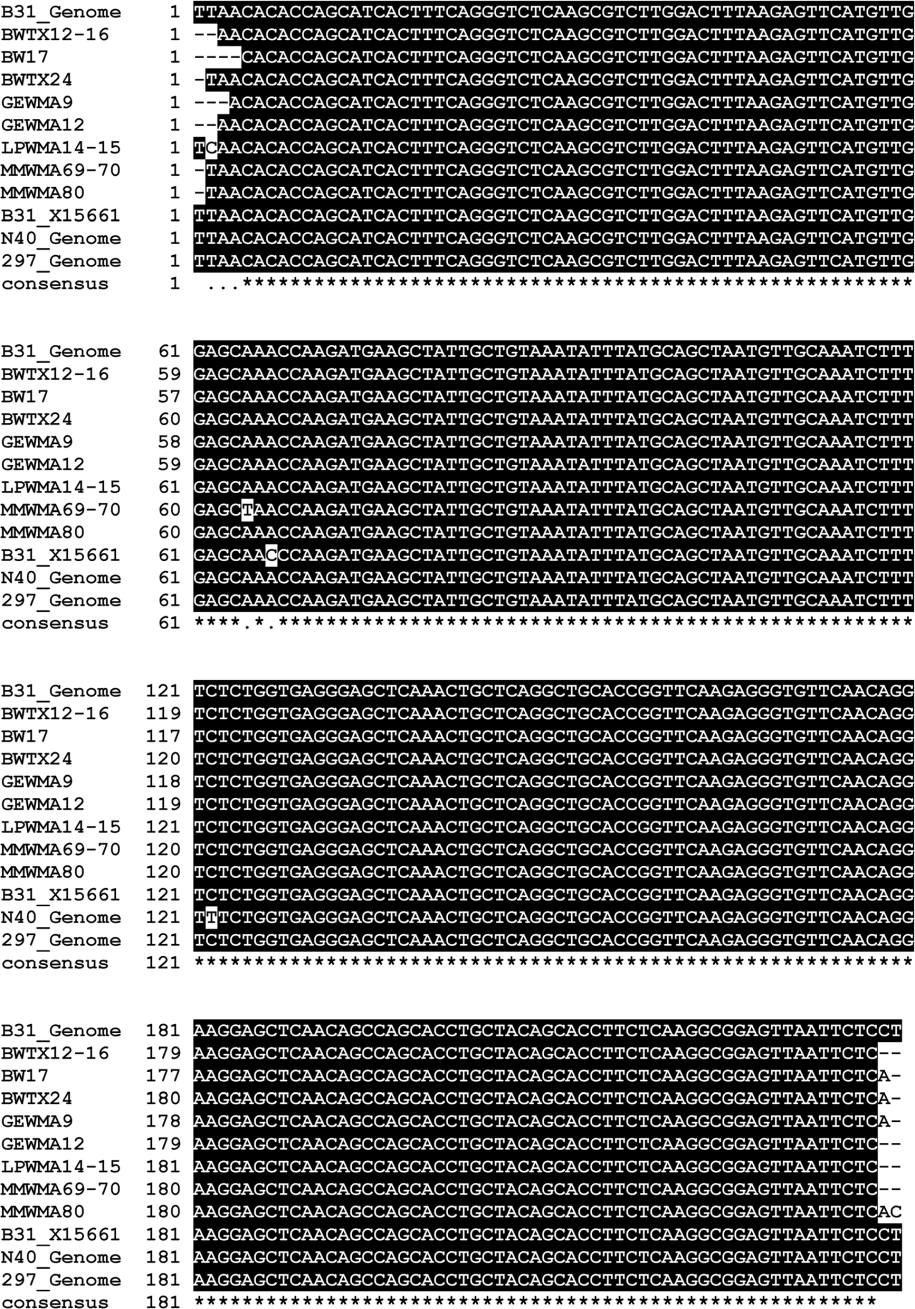
Alignment of *flaB* sequences. The *flaB* Texas specimen PCR product sequences obtained by Esteve-Gassent et al. [4] with the corresponding *flaB* regions of the *B. burgdorferi* B31 Genome (AE000783 and CP009656), the variant B31 *flaB* sequence X15661, the *B. burgdorferi* N40 Genome (CP002228), and *B. burgdorferi* 297 *flaB* (AB035616) are shown. The Texas specimen sequences are identical to the B31 sequence except for one nt difference at nt 64 in sample MMWMA69-70 and apparent sequence errors near the ends of other sequences. The location of the cytosine (C) difference reported by Esteve-Gassent et al. [4] is at nt 67 of the X15661 sequence. The GenBank accession numbers for the Esteve-Gassent et al. sequences are as follows (in parentheses): BWTX12-16 (KM875668.1); BW17 (KM875669.1); BWTX24 (KM875670.1); GEWMA9 (KM875671.1); GEWMA12 (KM875672.1); LPWMA14-15 (KM875673.1); MMWMA69-70 (KM875674.1); MMWMA80 (KM875675.1)

These results clearly demonstrate that the *flaB* sequences reported by Esteve-Gassent et al. [4] are indeed the same as the B31 sequence, and could have arisen from contamination of their samples from Texas with DNA from strain B31. Thus, their statement that ”*B. burgdorferi* B31 *flaB* has a cytosine (C) at position 75 in this alignment (Fig. one) while the Texas isolates had an adenine (A)” is incorrect. In fact, we have confirmed experimentally by Sanger sequencing that low-passage B31 contains an adenine at this position, in order to further rule out the possibility of a sequence error at this nucleotide. The *flaB* sequences are the only new information provided in the response by Esteve-Gassent et al. [4], yet these results only confirm that the Texas *flaB* sequences are the same as those in the positive control (strain B31). The *flaB* results in no way affect the interpretation of the IGS results, which are much more important because of the heterogeneity in IGS sequences observed among *B. burgdorferi* strains.

3. The reverse complement of the BWTX12-16 DNA sequence is mistakenly displayed in Fig. one

In this figure in the response from Esteve-Gassent et al. [4], the BWTX12-16 sequence exhibits a high number of nucleotide mismatches with the other sequences shown. Based on these differences, the authors state the following:

“BWTX12-16, a questing tick, has a significantly different sequence from either of the controls or the other Texan samples, suggesting that the degree of genetic variation of *B. burgdorferi* in the regions sampled likely exceed the values found by Feria-Arroyo *et al.* study [4], which only sampled a limited number of potential vertebrate hosts“.

However, Figure two of their response shows that the predicted amino acid sequence of BWTX12-16 is identical to those of the *B. burgdorferi* strains 297, N40 and B31, and most of the other Texas samples. Indeed, the reverse complement of the BWTX12-16 sequence was mistakenly used in Fig. one, resulting in the **appearance** of many nucleotide differences. In addition, the last 12 nucleotides of BWTX12-16 sequence in Fig. one do not match any sequence, including the BWTX12-16 sequence from GenBank that Esteve-Gassent et al. [4] had submitted. Because of these problems, the conclusion quoted above is not correct.

4. Figure two of the Esteve-Gassent et al. response reinforces sequence identity with B31 in most tick samples from Texas, but also indicates the presence of sequence errors in some of the sequences from Texas samples

With correction of amino acid 22 in the *B. burgdorferi* B31 sequence from “T” to “N”, most of the Texas samples have identical sequences to B31. However, two samples (GE64 and MM68) have extensive regions that are different from the FlaB sequence in *B. burgdorferi* strains. The GE64 nucleotide sequence has two frameshifts and MM68 has a single frameshift, which result in the aberrant amino acid sequences shown in Fig. two of Esteve-Gassent et al. [4]. Thus, these sequences contain sequence errors. The aberrant protein sequence shown for sample MM161 is not correct; its nucleotide sequence is identical to the B31 sequence, and encodes the same amino acid sequence. It appears that the MM68 amino acid sequence was inadvertently copied into the line for sample MM161, since the sequences are identical.

5. Figure three of the response by Esteve-Gassent et al. [4] inappropriately displays phylogenetic trees based on sequence errors and incorrect sequence orientation

Only nucleotide or amino acid sequences that cover the same range of sequence and are free from sequence errors can be utilized to construct reliable phylogenetic trees. In Fig. three-a of the response [4], both N40 and BWTX12-16 sequences were apparently included in the reverse orientation, resulting in a large, erroneous difference; this result is incongruous with their own Fig. two. In both Figs. three-a and -b, the heterogeneity shown is due to a combination of sequence errors and a failure to trim the sequences, so that they all encompass the exact same region. Thus, the results shown in Fig. three are not meaningful. The corrected phylogenetic tree (Fig. 2a) was prepared using the default algorithm at http://phylogeny.lirmm.fr/phylo_cgi/index.cgi and shows that all of the sequences from GenBank are identical, except for single nucleotide differences in the Texas sample MM69-10, the Wallich et al. *flaB* sequence X15661 and strain N40. The corresponding Figure from Esteve-Gassent et al. [4] is shown for comparison (Fig. 2b).

**Fig. 2:**
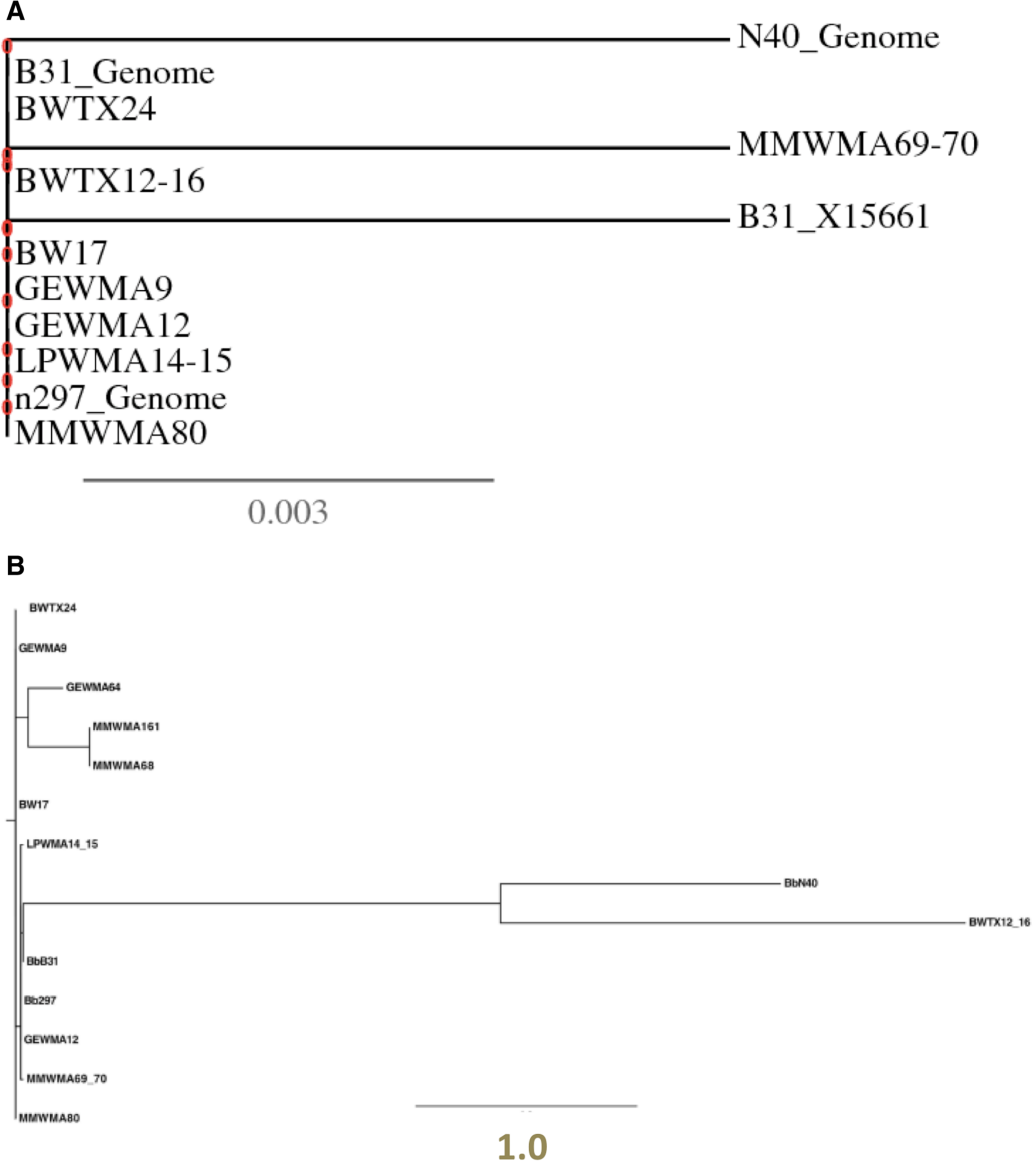
Comparison of phylogenetic trees. **a** Our reconstruction of the phylogenetic tree of *flaB* nucleotide sequences from 8 Texas specimens, the B31 genome, and the variant B31 *flaB* X15661. The tree was prepared using sequences trimmed to equal length by removing the first 4 positions and the last 2 positions of the aligned sequences in Fig. 1. The tree is consistent with 100 % identity between 7 of 8 Texas specimens with the B31 sequence. MMWMA69-10 and B31_X15661 each have one nucleotide difference. **b** Fig. three-a in the Esteve-Gassent et al. response [4], in which reverse complement sequences for *B. burgdorferi* N40 and BWTX12-16, as well as sequence ends that were not trimmed to the same length, were apparently utilized. Note the difference in the scale bars

6. The conclusions of Esteve-Gassent et al. [4] are contrary to all prior published reports regarding the heterogeneity of IGS sequences in *B. burgdorferi* strains

The following paragraph indicates their resistance to consider the possibility of DNA contamination and sequence errors in their results, with the conclusion that samples acquired from ticks feeding on white-tailed deer, gembok and dogs in a large geographical area harbour *B. burgdorferi* with identical or nearly identical IGS genotypes.

Esteve-Gassent et al. [4] state:

“Norris *et al*. stated in their letter that due to the low variability observed in the IGS from the Texas samples most, if not all of them, were likely to have been originated from the same clone which they assume could be the product of contamination with the B31 strain. We disagree with the interpretation put forward by Norris *et al*. and instead think it is more likely that the lack of variability reported in Feria-Arroyo *et al*. reflects the level of *B. burgdorferi* variability present in the Texas-Mexico transboundary region. Several of the ticks included in the Feria-Arroyo study were collected from white-tailed deer, gemsbok and dog. These mammalian hosts, particularly white-tailed deer, harbour ticks from several lineages. Thus, ticks collected from white-tailed deer, even if collected from the same individual, are likely to carry a representation of the *B. burgdorferi* strains present in a particular location. Thus, the *B. burgdorferi* genetic diversity reported by Feria-Arroyo *et al*., likely represents the genetic variation present in the Texas-Mexico transboundary region”.

Here, the authors provide no citations that indicate a similar sequence homogeneity among *B. burgdorferi* isolates in other geographical regions. They also do not address the overall high sequence identity of the IGS sequences (99.8 % to 100 %) and random distribution of sequence differences shown in Table one of the Letter to the Editor from Norris *et al.* [2]. In contrast, the corresponding IGS regions of representative strains B31, 297, N40, and SGE03-01 exhibit a lower sequence identity (95.2 % to 96.1 %) and clustered occurrence of sequence differences (Table two in [2]). In our previous Letter to the Editor [2], we had calculated that the probability of such low variability in the sequences obtained using the samples from Texas is less than 1.5 × 10^−17^. These points are addressed thoroughly in the prior Letter [2] and are thus not reiterated further here.

7. The article by Feria-Arroyo et al. [1] provides false and misleading information regarding the risk of *B. burgdorferi* infection in Texas and Mexico

To quote the Feria-Arroyo et al. article [1], “Infection with *B. burgdorferi* was detected in 45 % of *I. scapularis* ticks…”.

This degree of positivity is contrary to all prior Texas studies (except for one prior article by members of this group) and would indicate a tick infection rate as high as that found in the ‘hot spots’ of Lyme disease in the Northeastern United States (see [2] for further discussion regarding this point). Such a finding, if correct, would be alarming to the general public, public health agencies, physicians and scientists alike. Therefore, we strongly object to the following statement by Esteve-Gassent et al.

“Norris et al. suggest that the Feria-Arroyo et al. [4] publication is advocating a high LD risk in Texas and Mexico but this cannot be further from the truth”.

It is in fact a natural conclusion that a high tick infection rate connotes a heightened risk of human disease.

We conclude that, as in the case of the article by Feria-Arroyo et al. [1], the data and interpretations presented in the response from Esteve-Gassent et al. [4] are unreliable and are not valid scientifically. The Committee of Publication Ethics guidelines (http://publicationethics.org/files/retraction%20guidelines.pdf), to which BioMedCentral adheres, states that “Journal editors should consider retracting a publication if they have clear evidence that the findings are unreliable, either as a result of misconduct (e.g., data fabrication) or honest error (e.g., miscalculation or experimental error).” We believe that the latter assessment is the case. Therefore, we recommend the retraction of the Feria-Arroyo et al. article [1] from *Parasites & Vectors*.

## References

[1] Feria-Arroyo T, Castro-Arellano I, Gordillo-Perez G, Cavazos A, Vargas-Sandoval M, Grover A (2014). Implications of climate change on the distribution of the tick vector *Ixodes scapularis* and risk for Lyme disease in the Texas-Mexico transboundary region. Parasit Vectors..

[2] Norris SJ, Barbour AG, Fish D (2014). Diuk-Wasser MA. Analysis of the intergenic sequences provided by Feria-Arroyo et al. does not support the claim of high Borrelia burgdorferi tick infection rates in Texas and northeastern Mexico. Parasit Vectors..

[3] Burgdorfer W, Barbour AG, Hayes SF, Benach JL, Grunwaldt E, Davis JP (1982). Lyme disease, a tick-borne spirochetosis?. Science..

[4] Esteve-Gassent MD, Grover A, Feria-Arroyo TP, Castro-Arellano I, Medina RF, Gordillo-Perez G (2015). Prevalence of *Borrelia burgdorferi*-infected ticks from wildlife hosts, a response to Norris *et al*. Parasit Vectors..

[5] Kwok S, Higuchi R (1989). Avoiding false positives with PCR. Nature..

